# Assessment of anxiety-depression levels and perceptions of quality of life in adolescents with dysmenorrhea

**DOI:** 10.1186/s12978-018-0453-3

**Published:** 2018-01-26

**Authors:** Nilfer Sahin, Burcu Kasap, Ulviye Kirli, Nese Yeniceri, Yasar Topal

**Affiliations:** 1 Department of Child and Adolescent Psychiatry, Mugla Sitki Kocman University, School of Medicine, 48000 Mugla, Turkey; 2 Department of Obstetrics and Gynecology, Mugla Sitki Kocman University, School of Medicine, Mugla, Turkey; 3 Department of Pediatrics, Mugla Sitki Kocman University, School of Medicine, Mugla, Turkey; 4 Department of Family Medicine, Mugla Sitki Kocman University, School of Medicine, Mugla, Turkey

**Keywords:** Dysmenorrhea, Anxiety, Depression, Quality of life

## Abstract

**Background:**

This study aimed to assess the anxiety-depression levels and the perceptions of quality of life, as well as the factors affecting these variables, in adolescents with dysmenorrhea.

**Methods:**

The participants included 60 adolescents with dysmenorrhea and 41 healthy adolescents between the ages of 12 and 18. This study used the Pediatric Quality of Life Inventory (PedsQL) for assessing the perceptions of quality of life, the State-Trait Anxiety Inventory (STAI) for measuring anxiety levels, and the Children’s Depression Inventory (CDI) for measuring depression levels.

**Results:**

It was determined that compared to healthy controls, the depression and anxiety scores were higher and the quality of life was impaired in adolescents with dysmenorrhea. In addition, it was shown that the depression and anxiety levels increased and the psychosocial health subscale scores of quality of life decreased with increasing dysmenorrhea severity. However, the likelihood of dysmenorrhea was found to be higher with increasing depression scores, while the anxiety levels had no effect on dysmenorrhea.

**Conclusion:**

In dysmenorrhea management, it is important to enhance awareness among pediatric clinicians and gynecologists regarding the associations between dysmenorrhea and mental problems.

## Plain english summary

This study aimed to assess the anxiety-depression levels and the perceptions of quality of life, as well as the factors affecting these variables, in adolescents with dysmenorrhea.

The participants included 60 adolescents with dysmenorrhea and 41 healthy adolescents between the ages of 12 and 18. This study used the Pediatric Quality of Life Inventory (PedsQL) for assessing the perceptions of quality of life, the State-Trait Anxiety Inventory (STAI) for measuring anxiety levels, and the Children’s Depression Inventory (CDI) for measuring depression levels.

It was determined that compared to healthy controls, the depression and anxiety scores were higher and the quality of life was impaired in adolescents with dysmenorrhea. In addition, it was shown that the depression and anxiety levels increased and the psychosocial health subscale scores of quality of life decreased with increasing dysmenorrhea severity. However, the likelihood of dysmenorrhea was found to be higher with increasing depression scores, while the anxiety levels had no effect on dysmenorrhea.

In conclusion, in dysmenorrhea management, it is important to enhance awareness among pediatric clinicians and gynecologists regarding the associations between dysmenorrhea and mental problems.

## Background

Dysmenorrhea is a common gynecological problem characterized by pain at the abdominal and lumbar regions within the first few days of menstrual cycle, which limits daily activities of women. It is classified into two groups as primary and secondary dysmenorrhea. Primary dysmenorrhea is defined as cramps originating from the uterus during menstruation without any underlying pelvic pathology, while secondary dysmenorrhea is defined as menstrual pain resulting from underlying pelvic pathologies such as endometriosis [1]. Dysmenorrhea may be accompanied by headache, nausea, vomiting and fatigue. In adolescents, primary dysmenorrhea is seen in normal ovulatory cycles without any pelvic pathology. Its incidence has been reported as 60–93% in adolescents worldwide [2, 3], while studies in Turkey have reported an incidence of 55.5–70.0% among adolescents and young adults [4, 5].

Dysmenorrhea can affect the mental health and social life of an individual. Prior studies have shown that women with dysmenorrhea often fail to go to work or school, resulting in an impaired quality of life. The rate of absence days from school was reported to vary between 14% and 51% among women with dysmenorrhea [6]. Additionally, the rate of class attendance was reported to decrease by 29–50% during dysmenorrhea periods. Studies on adults report that a lower perception of quality of life among women with dysmenorrhea [7].

It is known that there is a close relation between pain and depression. The prevalence of headache or neck-shoulder pain is high in depression, while depression prevalence is high in patients with chronic pain syndromes [8]. Many studies have shown that depression enhances the effect of pain on social and occupational functionality and decreases the likelihood of response to medical therapy [9]. Moreover, anxiety levels were found to be higher in patients with migraine or other types of headaches [10]. It is thought that the levels of anxiety and depression, which have been found to be associated with many types of pain, are also associated with dysmenorrhea. Emotional and behavioral problems increase menstrual cycle problems and dysmenorrhea. A study on adolescents showed that anxiety, depression and smoking have an influence on dysmenorrhea [11]. Although advances have been achieved in the studies on the pathophysiology of dysmenorrhea, the association between gynecological and psychological factors has not yet been fully established. Currently, dysmenorrhea cannot be controlled in many cases, despite the availability of various treatments. Therefore, to achieve effective treatment, it is important to identify the factors that may affect the severity of dysmenorrhea and its comorbidities.

Previous studies have generally focused on adult women and there is a limited number of studies on the relationship between dysmenorrhea and mental problems, and its effect on the quality of life during adolescence. The present study aimed to assess the anxiety-depression levels and the perceptions of quality of life, as well as the factors affecting these variables, in adolescents with dysmenorrhea.

## Methods

The study included 60 adolescents between the ages 12 and 18 who presented at the Obstetrics and Gynecology and the Pediatric outpatient clinics of Muğla Sıtkı Koçman University, Faculty of Medicine, with dysmenorrhea between January 2015 and July 2015. Patients with mental retardation hampering communication and those with comorbid chronic diseases were excluded from the study. The control group included 41 adolescent girls of similar age and education level without any psychiatric and chronic disorders who were admitted at the Pediatric outpatient clinic.

All participants were asked to complete a demographic data sheet with questions on age, educational status, academic success, age at menarche, time from the onset of dysmenorrhea and duration of dysmenorrhea, family history of dysmenorrhea and measures taken during painful periods. The academic achievement levels of the cases were determined as good, modarate and bad, according to their declarations. The severity of dysmenorrhea was classified according to what they did for pain during the dysmenorrhea episodes (those who applied hot packs or used pain killer were evaluated as mild-moderate, those who applied emergency department were evaluated as severe dysmenorrhea). This study used the Pediatric Quality of Life Inventory (PedsQL) for assessing the perceptions of quality of life, State-Trait Anxiety Inventory (STAI) for measuring anxiety levels and Children’s Depression Inventory (CDI) for measuring depression levels.

The study was approved by the Ethics Committee on Scientific Research and Publications of Muğla Sıtkı Koçman University (approval date: 04.07.2014). All participants and their parents provided a written informed consent in accordance with the Helsinki Declaration.

### Data Collection Tools.

Pediatric Quality of Life Inventory (PedsQL): The scale was developed by Varni et al. in 1999 to measure the health-related quality of life of children and adolescents aged between 2 and 18 years [12]. The scale consists of seven forms including parent forms for children or adolescents aged 2–4, 5–7, 8–12 and 13–18 years, and self-report forms for children or adolescents aged 5–7, 8–12 and 13–18 years. The scale assesses the prior month of children and adolescents. It includes 23 items. Scoring is performed in three domains. Total score is obtained by summing the psychosocial health summary score and the physical health summary score, which in turn are obtained by adding the scores for the items assessing emotional, social and school functions. A higher total score indicates a better perception of the quality of life. The reliability and validity study for the Turkish version of the scale was conducted by Memik et al. [13].

Children Depression Inventory (KOVACS-CDI): This scale was developed by Kovacs in 1980 [14]. It is a self-report questionnaire including 27 items, and is applied to children and adolescents between the ages of 6 and 17. The cut-off value is 19 and the highest possible total score is 54 points. A higher total score indicates severe depression. It was adapted to Turkish by Oy in 1991 [15].

State-Trait Anxiety Inventory (STAI): This scale was developed by Spielberger [16]. The Turkish reliability and validity study of the scale was conducted by Öner et al. [17]. It consists of two scales, which are a trait-anxiety and a state-anxiety scale. Both scales include 20 items rated using a 4-point Likert scale. The trait-anxiety scale assesses how an individual generally feels, independently of the conditions involved. The state-anxiety scale assesses how an individual feels at a certain moment. The scores range from 20 to 80 in each scale. Higher scores indicate increased anxiety levels.

### Statistical analysis

Statistical analyses were performed using the SPSS for Windows version 20.0 (Statistical Package for Social Sciences Inc., Chicago, USA). The Student’s t test was used to compare the variables with normal distribution, while Mann Whitney U test was used to compare the data with skewed distribution. Chi-square test was used to compare categorical variables. Continuous variables were presented as descriptive statistics such as mean ± standard deviation. When assessing the associations between sociodemographic characteristics, clinical data and the scores of the patient and the control groups, the Pearson correlation analysis was used for parametric data, whereas Spearman’s correlation analysis was used for non-parametric data. To determine the effects of measures taken during menstrual period on scores, the Kruskal-Wallis test was used for the variables with normal distribution, whereas one-way ANOVA test was used for data with skewed distribution. Logistic regression analysis was used to assess the effects of scores on dysmenorrhea. A *p* value < 0.05 was considered as statistically significant.

## Results

In our study, the mean age was 15.46 ± 1.38 in the dysmenorrhea group and 15.24 ± 1.47 in the control group. The mean age at menarche was 12.65 ± 1.13 in the dysmenorrhea group and 12.75 ± 0.85 in the control group. When educational status was considered, it was observed that 78.3% of the patients in the dysmenorrhea group were at high school, while 21.7% were at secondary school. In the control group, 65.9% of the patients attended high school, while 34.1% were at secondary school. When academic success was considered, it was found that 61.7% of the patients in the dysmenorrhea group reported as having good academic success, while 38.3% reported moderate academic success. In the control group, 48.8% reported good academic success, while 51.2% reported moderate academic success. When the time from the onset of dysmenorrhea was evaluated in the dysmenorrhea group, it was found to be 23.68 ± 15.90 months, while the mean duration of dysmenorrhea was determined as 2.56 ± 1.49 days. In the dysmenorrhea group, there was history of dysmenorrhea in 63.3% of the patients with dysmenorrhea in the mother in 48.3% and in sister in 15.0%. When measures taken against dysmenorrhea were evaluated, it was found that 53.3% (*n* = 32) have taken painkillers, that 28.3% (*n* = 11) have visited emergency service, and that 18.3% (n = 11) have used hot packs. In the dysmenorrhea group, 41.7% had a day of absence from school. There were no statistically significant differences between the patient and control groups in terms of demographic data (*p* > 0.05).

Based on the scores obtained from the data collection tools, the distribution of the PedsQL, STAI-T STAI-S scores were found normal, while physical health and psychosocial health summary scores showed a skewed distribution. The mean score of total PedsQL was 63.60 ± 8.98 in the dysmenorrhea group and 79.67 ± 9.37 in the control group (p: 0.000). The median value of physical health summary score of PedsQL in the dysmenorrhea group was 62.50, while in the control group it was 75.62 (p:0.000). The median value of psychosocial health summary score of PedsQL in the dysmenorrhea group was 66.66, while in the control group this value was 83.33 (p:0.000). The mean score of STAI-T in the dysmenorrhea group was 38.18 ± 11.15, while this value was 32.46 ± 6.30 in the control group (p:0.004). The mean score of STAI-S in the dysmenorrhea group was 47.70 ± 10.00. In the control group, this value was 40.36 ± 6.80 (p: 0.024). The median score of CDI was determined as 14 in the dysmenorrhea group, which was 8 in the control group (p:0.000). The differences between the two groups in terms of scale scores were statistically significant (Table 1).

**Table 1:** Scores in dysmenorrhea and control groups

	Dysmenorrhea group Mean ± SD Median (min-max)	Control group Mean ± SD Median (min-max)	*p*
Total PedsQL	63.60 ± 8.98	79.67 ± 9.37	0.000
PHSS	62.50 (15.62–81.25)	75.62 (53.75–100)	0.000
PSHSS	66.66 (41.60–83.33)	83.33 (60–98.33)	0.000
STAI-T	38.18 ± 11.15	32.46 ± 6.30	0.004
STAI-S	47.70 ± 10.00	40.36 ± 6.80	0.024
CDI	14 (4–36)	8 (3–20)	0.000

*PedsQL* Pediatric Quality of Life Inventory, *PHSS* Physical health summary score, *PSHSS* Psychosocial health summary score, *STAI-T* Trait anxiety inventory, *STAI-S* State anxiety inventory, *CDI* Children Depression Inventory, *p* significance value

When the correlation of age, age at menarche, time from the onset of dysmenorrhea and duration of dysmenorrhea with the scale scores was examined in the dysmenorrhea group; it was found that the scale scores had no correlation with age, age at menarche and time from the onset of dysmenorrhea (*p* > 0.05), while the total PedsQL score was negatively correlated with the duration of dysmenorrhea (r:-0.284, p:0.028), and the CDI was positively correlated with the duration of dysmenorrhea (r:0.255, p: 0.049) (Table 2).

**Table 2:** Associations between scores and demographic variables in the dysmenorrhea group

		Age	Age at menarche	Time from onset of dysmenorrhea	Duration of dysmenorrhea
Total PedsQL	R	−0.26	−0.46	0.06	−0.284
	P	0.843	0.727	0.649	**0.028**
PHSS	R	−0.57	−0.111	0.204	−0.137
	P	0.666	0.399	0.119	0.296
PSHSS	R	−0.052	0.008	−0.12	−0.214
	P	0.692	0.954	0.927	0.100
CDI	R	0.114	0.097	−0.070	**0.255**
	P	0.255	0.336	0.593	**0.049**
STAI-T	R	0.141	0.149	0.033	0.234
	P	0.160	0.138	0.803	0.072
STAI-S	r	0.140	0.153	−0.140	0.239
	p	0.162	0.127	0.286	0.066

Spearman-Pearson correlation analysis

*PedsQL* Pediatric Quality of Life Inventory, *PHSS* Physical health summary score, *PSHSS* Psychosocial health summary score, *STAI-T* Trait anxiety inventory, *STAI-S* State anxiety inventory, *CDI* Children Depression Inventory, *p* significance value, *r* correlation coefficient

When scores were considered according to the measures taken during dysmenorrhea, a significant difference was found in scores other than the total and physical health summary scores of PedsQL in cases visiting emergency department compared to those using painkillers or hot packs (Table 3).

**Table 3:** Associations between scores and measures taken during dysmenorrhea

	Measures taken during dysmenorrhea	Mean ± SD	F	*p*
Total PedsQL	Emergency department visit	60.17 ± 8.75	1.857	0.164
	Pain killer	65.27 ± 9.21		
	Hot packs	64.05 ± 7.804		
PHSS	Emergency department visit	63.23 ± 12.90	0.727	0.468
	Pain killer	62.01 ± 12.06		
	Hot packs	57.23 ± 17.72		
PSHSS	Emergency department visit	58.99 ± 11.74	4.143	**0.021**
	Pain killer	67.26 ± 8.98		
	Hot packs	67.47 ± 10.47		
CDI	Emergency department visit	24.11 ± 6.35	38.49	**0.000**
	Pain killer	12.15 ± 3.52		
	Hot packs	11.54 ± 5.42		
STAI-T	Emergency department visit	47.00 ± 11.07	9.986	**0.000**
	Pain killer	34.00 ± 9.31		
	Hot packs	36.72 ± 8.85		
STAI-S	Emergency department visit	55.05 ± 8.45	8.215	**0.001**
	Pain killer	44.25 ± 8.80		
	Hot packs	46.36 ± 10.16		

*PedsQL* Pediatric Quality of Life Inventory, *PHSS* Physical health summary score, *PSHSS* Psychosocial health summary score, *STAI-T* Trait anxiety inventory, *STAI-S* State anxiety inventory, *CDI* Children Depression Inventory, *p* significance value

When effects of depression and anxiety on dysmenorrhea were considered, the STAI-T and STAI-S scores were found to have no effect on dysmenorrhea, while one-point increase in CDI score caused an increase in the likelihood of dysmenorrhea by 0.194 (p:0.006) (Table 4).

**Table 4:** Effects of CDI, STAI-T ad STAI-S on likelihood of dysmenorrhea

	β	*p*
CDI	0.194	**0.006**
STAI-T	−0.019	0.928
STAI-S	0.027	0.499

*CDI* Children Depression Inventory, *STAI-T* Trait anxiety inventory, *STAI-S* State anxiety inventory, *p* significance value

## Discussion

Our study, which assessed depression and anxiety levels as well as perceptions of quality of life in adolescents with dysmenorrhea, found that, compared to healthy controls, the depression and anxiety scores were higher and the quality of life was lower in adolescents with dysmenorrhea. In addition, it was shown that the depression and anxiety levels increased and the psychosocial health subscale scores of quality of life decreased with increasing severity of dysmenorrhea. While the likelihood of dysmenorrhea was found to increase with increasing depression scores, it was observed that anxiety levels had no effect on dysmenorrhea.

In our study, no significant difference was identified between the dysmenorrhea and control groups with respect to the age at menarche and academic success. There are a number of studies indicating a correlation between age at menarche and dysmenorrhea; however, there are also studies indicating no significant differences [18, 19]. It is known that dysmenorrhea decreases academic success by causing days of absence from school. Our study found no significant difference in academic success, although, compared to the control group, the scores of psychosocial health summary and quality of life were significantly lower in adolescents with dysmenorrhea. In dysmenorrhea group, academic success was expected to be poorer compared to the control group, since the rate of absence days from school was 41.7% in this group. Although our study is in agreement with previous studies indicating that dysmenorrhea causes days of absence from school [20], our study differs from those reporting that dysmenorrhea is associated with decreased academic success. It is believed that this difference might have stemmed from the fact that we used self-assessments for evaluating academic success, rather than objective data such as grade average. This, in fact, represents a limitation of the current study.

Many studies have shown that menstrual symptoms are associated with mental status. In a recent study, depression, aggression, insomnia, daytime sleepiness and sleep apnea scores were significantly higher in the dysmenorrhea group than in normal controls [21]. In a study with 400 post-menarche women between the ages of 14 and 20, anxiety and depression scores were found higher in women with dysmenorrhea [22]. It is thought that dysmenorrhea disrupts healthy physical and mental development in adolescence by impairing the quality of life with increased days of absence from school [11], although the mechanism underlying the relationship between dysmenorrhea and psychological problems has not yet been fully elucidated. The association of anxiety and depression with dysmenorrhea can be explained as the adverse effect of chronic pain on mental health. It is known that psychiatric problems, mainly depression, are more common in patients with chronic pain. A study investigating the relationship between personality traits and dysmenorrhea in 49 women found that higher neuroticism-anxiety and depression levels were associated with dysmenorrhea. The same study also emphasized that chemicals such as prostaglandins, vasopressin and phospholipids released during menstrual pain are also associated with depression and anxiety [23]. In agreement with previous studies, our study identified increased levels of depression and anxiety. In addition, depression was found to have a more prominent effect on dysmenorrhea than anxiety when the effects of depression and anxiety on dysmenorrhea were considered, suggesting that adolescents with higher depression scores are predisposed to dysmenorrhea. The finding of increased depression and anxiety levels in cases with dysmenorrhea leads to two questions: whether dysmenorrhea itself impairs an individual’s mental health by affecting social life, or whether dysmenorrhea is more commonly seen in individuals with higher levels of anxiety and depression. In our study, the finding of higher anxiety and depression scores in cases with dysmenorrhea suggests that dysmenorrhea itself impairs mental health; however, when the presence of dysmenorrhea was assessed in cases with high depression and anxiety scores, the likelihood of dysmenorrhea was found to be higher in cases with high depression scores. In other words, it was shown that the likelihood of dysmenorrhea was not more common in cases with high anxiety levels. This finding is inconsistent with previous studies reporting a significant effect of anxiety on pain [24].

The studies assessing the perception of quality of life in women with dysmenorrhea have shown that dysmenorrhea impairs the perception of quality of life. In our study, all scores from the quality of life scales were lower in cases with dysmenorrhea compared to the control group. In a study on 623 university students from Turkey, Ünsal et al. evaluated the incidence of dysmenorrhea and the perception of quality of life. Authors showed that the quality of life was impaired by increased severity of dysmenorrhea [7]. In another study that assessed the quality of life during the menstrual period, where there were complaints of dysmenorrhea, and during the follicular phase, where there was no pain in women with dysmenorrhea, it was determined that the perception of quality of life was impaired during the menstrual period [25]. Although the perception of quality of life was found to be poorer in the dysmenorrhea group compared to the control group in our study, it will be more valuable to assess the quality of life during dysmenorrhea and the interictal period in a way similar to the abovementioned study in order to determine the effects of dysmenorrhea on the quality of life, given that the duration of dysmenorrhea is generally 2–3 days with a longer interictal period. Our study determined that psychosocial health summary score of PedsQL was lower in cases visiting emergency department during dysmenorrhea compared to those using painkillers or hot packs. This suggests that the severity of dysmenorrhea affects the quality of life. In our study, anxiety and depression scores were also found higher in cases visiting emergency department during dysmenorrhea compared to those using painkillers or hot packs. In other words, the anxiety and depression scores increased with increasing severity of pain. It is anticipated that increased severity of pain will lead to negative effects on mental health which, in turn, would lead to increased days of absence from school, poorer academic success and impaired quality of life.

Nonetheless, there are certain limitations to this study. Its cross-sectional design and small sample size are the main limitations. We believe that further studies with larger sample size are needed to clarify the relationship between mental health and dysmenorrhea. The lack of the psychiatric examination and a semi-structured interview was evaluated as one of the major limitations of our study.

## Conclusion

In conclusion, it was determined that, compared to the healthy controls, the anxiety and depression levels were higher and the perception of the quality of life was lower in adolescents with dysmenorrhea. It was found that increasing severity of dysmenorrhea further increased the extent of impairment in mental health and the perception of psychosocial health. The likelihood of dysmenorrhea was shown to be higher among cases with higher levels of depression. In dysmenorrhea management, it is important to enhance awareness among pediatric clinicians and gynecologists regarding the associations between dysmenorrhea and mental problems. It is believed that better results can be achieved in the management of dysmenorrhea cases, and that the quality of life can be further improved, by referring the child to a psychiatrist whenever necessary and through psychiatric follow-up.

## Abbreviations

CDI: Children Depression Inventory
PedsQL: Pediatric Quality of Life Inventory
PHSS: Physical health summary score
PSHSS: Psychosocial health summary score
STAI-S: State anxiety inventory
STAI-T: Trait anxiety inventory
